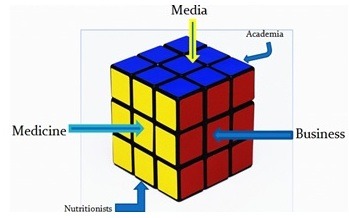# Understanding Health Food is like Rubik's Solving one side of the problem does not actually bring you closer to a total solution

**Published:** 2014

**Authors:** Stanton John L.

**Affiliations:** * Saint Joseph’s University, Philadelphia, USA

**Abstract**

**When considering what health food is, we have many different “faces to solve”**

- We have the medical community telling us what is good and bad for our health

- We have nutritionists telling us what not to eat and what to eat

- We have food companies putting all sort of things in advertising and on the packaging

- We have academic researchers employing PR firms to broadcast their most recent conclusions

- We have the media a willing partner to give any headline to any story that might garner readers’ attention.

- Public interest groups attacking anyone that does not promote brown beans and rice

- And of course the government, which tries to stand above the fray an act like the unbiased interpreter of all the above. 

**Keywords:**health food, Rubik Cube, obesity, fat

**We are getting fatter**

**Figure:**
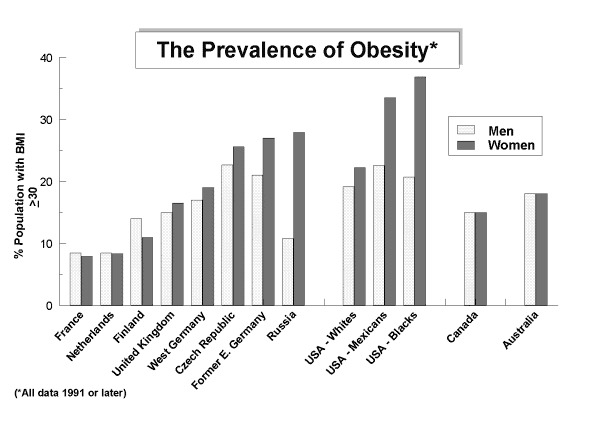


**Figure:**
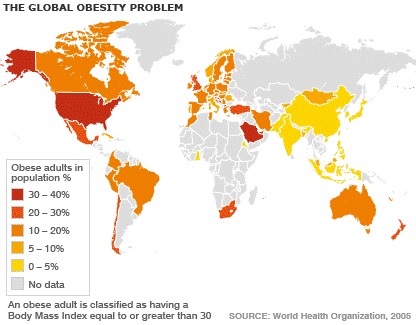


Romania appears to have about a 15% obesity rate

**So what!**

According to study data, more than half a billion men and women (nearly one in nine adults) are clinically obese, while according to 2008 statistics, nearly one woman in seven and one man in 10 were found obese. The Lancet reported, “It is expected to cost tens of millions of preventable deaths unless rapid and widespread actions are taken by governments and health-care systems worldwide." It cost lives and it cost money.

**Diabetes is increasing**

An estimated 350 million people worldwide have diabetes, according to new research published in the Lancet. It describes the disease as a “rising global hazard” and says global diabetes rates have doubled from 1980 to 2008.

**Cut fat from your diet. We all no fat is Evil, right?**

Fat is not a four-letter word.

For many people, straightening out the facts about fat has been tough. Long demonized -- 20 years ago, many grocery shelves were packed with nonfat and low-fat products -- *mono- and polyunsaturated fats are now embraced for their health benefits.*

Research has shown that these fats help improve blood cholesterol levels, either by cutting levels of low-density lipoprotein, one of the most damaging forms of cholesterol, or by boosting levels of high density lipoprotein, a protective type of cholesterol. The 2005 Dietary Guidelines made fat's revised role official, advising that a healthy intake could range from 20 percent of daily calories up to as much as 35 percent.

**But Why You Should Avoid "Fat-Free" Foods**

During the past several decades, reduction in fat intake has been the focus of national dietary recommendations. In the public's mind, the words "dietary fat" have become synonymous with obesity and heart disease, whereas the words "low-fat" and "fat-free" have been synonymous with heart health.

It is now increasingly recognized that the low-fat campaign has been based on little scientific evidence and may have caused unintended health consequences. -- from "Types of Dietary Fat and Risk of Coronary Heart Disease: A Critical Review;" Frank B. Hu, *Journal of the American College of Nutrition*, Vol. 20 (2001).

**We should eat more Fish?? Less Red meat and more Fish**

**Figure:**
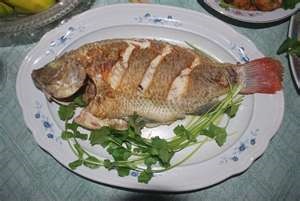


In 2004, the FDA issued seafood advisories for children under 12, pregnant and nursing women, and women who might become pregnant, suggesting they avoid shark, swordfish, tilefish, and king mackerel.

The warning filtered into the general public's consciousness, and *almost a third of Americans incorrectly came to believe the advice applied to everyone* and extended to all species of fish, according to a survey by the Center for Food, Nutrition, and Agricultural Policy.

**Organic, Local or Vegan**

- We read a lot about how good organic is for you. However can the world agriculture support a significant increase in organic

- Only about 0.9% of global acres is organic

- a prominent 21-year Swiss study found an average of 20% lower organic yields over conventional

**Is salt bad for you???**

The Cochrane Review of Sodium and Health: There is still insufficient power to exclude clinically important effects of reduced dietary salt on mortality or cardiovascular disease (CVD).

Government’s Anti-Salt Agenda Violates Law, Treats 300 Million Americans Like Lab Rats in Risky Trial ….Salt Institute

**However, USDA says**

Over 30 years of scientific evidence shows that a diet containing more than 6 grams of salt per day (2,400 milligrams of sodium----the amount in a little more than a teaspoon of salt) is associated with elevated blood pressure. Increased blood pressure can lead to hypertension, heart disease, stroke, and kidney disease. It is important to note that elevated blood pressure can harm the body before symptoms occur.

**The media??**

- Our worse enemy or just doing their job?

- Diabetes again linked to colon cancer risk 

- Edible Healing: Food Cures for Cancer

- Cancer Cure: Raw Foods?

- Beating Cancer - Wonder Foods Believed to Cure Cancer

- The objective of media is to get people to BUY newspapers or watch TV or to read their website.

- The need breakthroughs but science is not about breakthroughs they are about the convergence of studies and the replication of studies…that takes time.

**What must we do?**

- To begin if we expect the global community to be healthier we must:

- Provide guidelines that are consistent with communities.

- For example, suggestions that require people to cook more shop more or prepare more will (and have failed).

- The US is in a Time Famine.

- Have messages that are consistent and based on replicated science. We must stop the flood of single studies in clinical settings that make grandiose promises of health outcomes. Even if results appear promising, there must be more convergence of research to make claims.

- Insist on Multi-disciplinary research teams 

- For example on a recent USDA grant on Mushrooms and Vitamin D we had food marketers, Agricultural Economists, Food Processors, and nutritionists.

- Telling US consumers to eat beans and Rice when they barely have time to open a can makes no sense.

- Food companies must:

- Invest in new science….provide firms incentives to conduct this research (a la patents)

- Help monitor false claims – whistleblowers

- Reformulate foods to meet healthier standards

- Nutritionist must:

- Learn about science

- Recognize what we don’t know

- Not declare as fact relationships still uncertain

- Governments must:

- Clarify (internationally) well understood links between diets and health, be clear what messages are allowed and act against those firms violating these messages

- Educate children how to cook, eat and farm

- Subsidize healthy behavior (fruits and veg, gym membership) rather than penalize unhealthy (fat/sugar taxes)

- Medical Community

- Learn Nutrition 

- Media must:

- Develop guidelines to report on nutrition and health

- Use the same fact checking as other types of stories

- Try to present all sides of the research

**The solution**

**Figure:**